# Cell-Free Synthetic Biology Platform for Engineering Synthetic Biological Circuits and Systems

**DOI:** 10.3390/mps2020039

**Published:** 2019-05-14

**Authors:** Dohyun Jeong, Melissa Klocke, Siddharth Agarwal, Jeongwon Kim, Seungdo Choi, Elisa Franco, Jongmin Kim

**Affiliations:** 1 Division of Integrative Biosciences and Biotechnology, Pohang University of Science and Technology, 77 Cheongam-ro, Pohang, Gyeongbuk 37673, Korea; gyu9506@postech.ac.kr (D.J.); jeongwon96@postech.ac.kr (J.K.); choisd@postech.ac.kr (S.C.); 2 Department of Mechanical Engineering, University of California at Riverside, 900 University Ave, Riverside, CA 92521, USA; klocke@ucr.edu (M.K.); sagar002@ucr.edu (S.A.); 3 Department of Mechanical and Aerospace Engineering, University of California at Los Angeles, 420 Westwood Plaza, Los Angeles, CA 90095, USA

**Keywords:** synthetic biology, cell-free transcription-translation, rapid prototyping, artificial cell, riboregulator, DNA origami, mathematical model

## Abstract

Synthetic biology integrates diverse engineering disciplines to create novel biological systems for biomedical and technological applications. The substantial growth of the synthetic biology field in the past decade is poised to transform biotechnology and medicine. To streamline design processes and facilitate debugging of complex synthetic circuits, cell-free synthetic biology approaches has reached broad research communities both in academia and industry. By recapitulating gene expression systems in vitro, cell-free expression systems offer flexibility to explore beyond the confines of living cells and allow networking of synthetic and natural systems. Here, we review the capabilities of the current cell-free platforms, focusing on nucleic acid-based molecular programs and circuit construction. We survey the recent developments including cell-free transcription–translation platforms, DNA nanostructures and circuits, and novel classes of riboregulators. The links to mathematical models and the prospects of cell-free synthetic biology platforms will also be discussed.

## 1. Introduction

Synthetic biology focuses on engineering biological circuits to manipulate biological systems and technological applications. Formative works in synthetic biology demonstrated the creation of simple regulatory circuits in *Escherichia coli* [1,2]. The dynamics of these synthetic circuits were reasonably captured through mathematical modeling, driving further developments of forward-engineering approaches [3]. As the scope of synthetic biological circuits increases dramatically, comprehensive design, analysis, and predictive modeling in cellular contexts becomes challenging despite progress in computer-aided designs [4,5]. Cell-free synthetic biology provides a paradigm to test components and circuits in a well-controlled environment that is similar to physiological conditions [6]. Cell-free approaches could expedite development and exploration of synthetic system designs beyond the confines of living organisms. In turn, novel, sustainable, and cost-effective technologies based on cell-free synthetic biology could help meet broader, worldwide challenges in the future.

In this article, we review the current scope of cell-free synthetic biology, focusing on synthetic circuits and systems using nucleic acid-based programs. We limit ourselves to the design and applications of these synthetic molecular circuits. Readers are referred to other excellent reviews for recent developments in other areas of cell-free synthetic biology such as cell-free metabolic engineering [7,8]. We first survey cell-free transcription–translation platforms that are gaining popularity as a testbed for rapid prototyping of synthetic circuit elements and circuitry. We then review in vitro model dynamical systems and recent progress in de novo-designed RNA regulatory toolkits for synthetic biology. Next, we discuss synthetic cell approaches through compartmentalization and the prospect of nucleic acid-based nanostructures and circuits to function in cell-like environments. Finally, we discuss modeling approaches and developments as well as their links with the future of synthetic biological circuits.

## 2. Cell-Free Transcription–Translation Platform for Synthetic Biology

**Figure 1:**
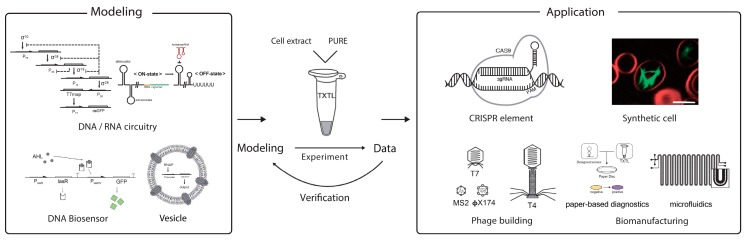
Overview of the cell-free transcription–translation platform. The cell-free transcription–translation platform including *Escherichia coli* cell-extract (TXTL) system and PURE system, allows for the prototyping of synthetic circuits rapidly through iterative cycles of experiments and computational modeling. TXTL has a number of applications, such as characterization of CRISPR elements or construction of synthetic cells. Reproduced with permission from [15,16].

Synthetic biology approaches for achieving novel and complex functionality in cellular systems have shown significant progress. Using cells as chassis to engineer circuits, however, presents challenges for rapid design–build–test cycles despite ongoing development of applicable tools. The cell-free transcription–translation system presents an attractive alternative to construct, characterize, and interrogate synthetic biological circuits (Figure 1). Although a number of cell-free expression systems have been developed, including rabbit reticulocytes, wheat germ, and insect cells, the prokaryotic extract cell-free expression system is the most popular and is commercially available [9]. We will mainly discuss the *E. coli* cell-extract system, termed as ‘TXTL’ in this section [10]. Compared to in vivo systems, the cell-free TXTL platform enables rapid prototyping of genetic circuit design using either generic plasmid DNA templates or short linear DNA templates [11,12]. Further, because TXTL-based circuits are implemented in vitro, these circuits are not limited by production of toxic proteins and chemicals or use of unnatural amino acids, which limit implementation of the same circuits in living cells [13,14].

The TXTL platform is not without limitations and challenges. Energy sources can be easily depleted in batch mode [17], while enzymes can degrade nucleic acids and protein products within the cell extract. Additionally, a complete understanding of machinery in TXTL system has yet to be achieved, and the yields of TXTL systems can be less than yields of corresponding in vivo systems [6]. Molecular crowding effects [18] or unintentional crosstalk between components [19] could contribute to these issues.

The PURE system is a completely purified cell-free expression platform containing the T7 RNA polymerase (RNAP) with fewer active components than cell extract-based TXTL systems [20]. In principle, the concentration of individual components can be adjusted in the PURE system during reconstitution, and purification of output proteins is straightforward by using affinity chromatography. The PURE system is costly and typically has a smaller yield than TXTL, but it can be advantageous for applications that require clear background and long-term storage of genetic elements [21].

The unique advantages of cell-free reactions make TXTL an ideal platform for prototyping genetic circuits by characterizing the properties and activities of circuit components [22]. For instance, the behaviors of CRISPR components (gRNA, protospacer adjacent motif (PAM) sequence, Cas9, and inhibitors of Cas9) are characterized using TXTL [23]. Importantly, the design–build–test cycle can be performed much faster than in vivo systems, thereby facilitating rapid prototyping of engineered circuits [24,25,26]. Circuit elements characterized in TXTL can be ported to an in vivo system, as demonstrated by the three- and five-node oscillator systems characterized in TXTL and successfully ported to *E. coli* [27].

**Figure 2:**
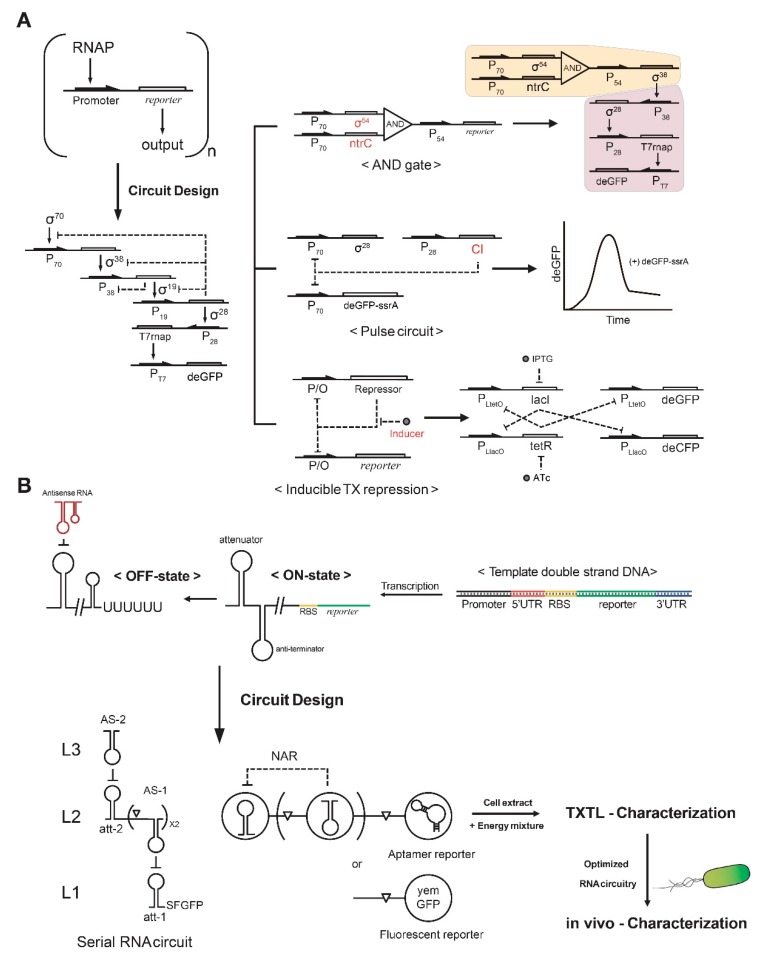
Systematic construction of DNA and RNA circuitry in TXTL. (**A**) Basic (input–parameter–output) modules are integrated to build complex synthetic circuits. Assembly of an AND gate, repressor, and inducer modules provides versatile and scalable circuits for synthetic biology applications. (**B**) RNA regulatory motifs are utilized for synthetic circuits such as a serial RNA circuit and a negative autoregulation circuit (NAR). The RNA circuits can be optimized in TXTL and ported to in vivo conditions.

Early works by Noireaux and colleagues demonstrated a multistage cascade circuit by superimposing several basic (input–parameter–output) units [11], where bacteriophage RNAP drove the expression of cascade circuits. However, with cascades including three to five stages, circuit performance was limited because of simple regulatory structures and extensive resource utilization. To expand the repertoire of transcriptional regulatory elements, sigma factors and cognate *E. coli* promoters were used for circuit construction [10,22]. They were able to demonstrate a five-stage transcriptional activation cascade through clever use of the different affinities of sigma factors to the core RNAP for efficient signal propagation (Figure 2A, left). Regulatory functions can be expanded by integrating various regulatory elements for constructing a more complex circuit (Figure 2A, right). Simultaneously monitoring the concentrations of produced RNA and proteins can assist in debugging synthetic circuits characterized in TXTL [19].

Synthetic RNA circuits are also efficiently and easily characterized in TXTL. Networks constructed from riboregulators propagate signals directly as RNAs, thus bypassing intermediate proteins, making these networks potentially simpler to design and implement than transcription factor-based layered circuits [28]. qPCR and next-generation sequencing techniques can characterize species, structural states, and interactions of RNAs [29]. Since the speed of signal propagation within circuits is determined by the decay rate of the signal, RNA networks can operate on much faster time scales than protein networks [30]. An early model of an RNA circuit used the transcriptional attenuator structure of RNA and its complementary antisense RNA [28]. The hairpin structure of the transcriptional attenuator was targeted by antisense RNA, which promoted the formation of a downstream intrinsic terminator hairpin that caused RNAP to fall off and stop transcription (Figure 2B). Other simple RNA-based circuits have also been characterized in TXTL systems [26,31], such as a negative autoregulation circuits, which use the attenuator and antisense RNA simultaneously [32].

The strength of the TXTL platform enables the expression of remarkably large natural DNA programs. A large amplification of the T7 phage, with a genome size of 40 kbp and supplemented with thioredoxin, was observed in vitro [33]. Cell-free self-organization of the even larger T4 phage, with a genome size of 169 kbp, under in vitro conditions was observed by increasing molecular crowding effects [34]. Replication of viral genomes occurred simultaneously with phage gene expression, protein synthesis, and viral assembly.

Beyond scientific inquiry, several practical tools emerged for using cell-free expression platforms. For instance, sequence-specific colorimetric detection of Zika viral RNA can be performed at single-base resolutions through a cell-free reaction on a paper disc. This paper-based diagnostic platform is advantageous because it is mobile and low-cost [35]. Another recent development demonstrated microfluidic reactors [15] and paper-based devices [36] that produced therapeutic proteins on demand.

## 3. In Vitro Synthetic Gene Circuits

In vitro regulatory networks are model systems that offer a flexible test bed for the design principles of biochemical networks without the complexity of cellular environments. In vitro regulatory models can be stripped of cellular machinery for protein translation and may use nucleic acid-based programs to design biochemical networks. In this section we will discuss simplified in vitro synthetic regulatory models using the synthetic transcription-based genelet system as an example [37].

**Figure 3:**
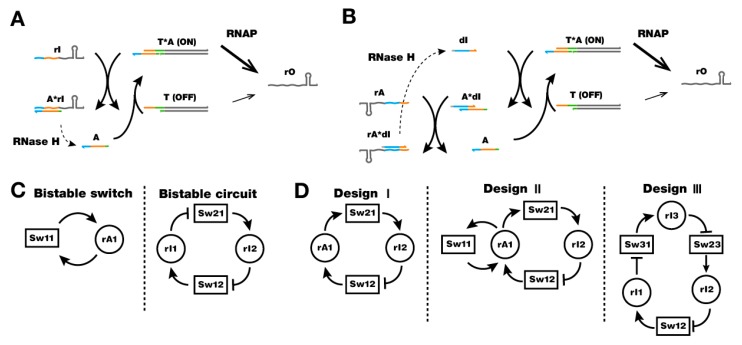
Genelet switches and circuits. (**A**) Design and operation mechanism of an inhibitable switch. The input, RNA inhibitor, sequesters the DNA activator from the active template and turns the switch to an OFF state. (**B**) Design and operation mechanism of an activating switch. The input, RNA activator, strips off DNA inhibitor bound to DNA activator. The released DNA activator in turn can turn the switch to an ON state. The sequence domains are color coded to indicate identical or complementary sequences. (**C**) Schematics of bistable circuits. A single switch with positive autoregulation (left) or two mutually inhibiting switches (right) can show bistability. (**D**) Schematics of oscillators. An activating switch and an inhibiting switch (Design I), Design I with further positive-autoregulation (Design II), and three inhibiting switches in a ring (Design III) form an overall negative feedback to achieve oscillation. Reproduced with permission from [15,16].

Genelets are synthetic DNA switches that form a partially double-stranded (ds) DNA template. The expression states of a genelet are controlled by specific DNA inputs, which are recognized by an incomplete promoter region in the template [38,39,40]. The genelet system consists of synthetic DNA templates and two enzymes: T7 RNAP and *E. coli* ribonuclease H (RNase H). Because of the incomplete, partially single-stranded (ss) promoter region of genelets, the DNA template (‘T’) by itself is poorly transcribed [41]. An ssDNA activator (‘A’) can bind to complete the promoter region, and the resulting complex (‘T-A’) transcribes well, approximately half as efficiently as a full dsDNA template [39]. The activity of genelets can be controlled by nucleic acid inputs forming an inhibitory regulation [39] and an excitatory regulation [42]. The inhibitable switch is turned off by an RNA inhibitor that binds to DNA activator more favorably than the switch template thereby removing the activator from the template (Figure 3A). The activating switch is turned on by an RNA activator that binds to a DNA inhibitor and releases the DNA activator (Figure 3B). Both the DNA inhibitor and activator contain a ‘toehold’, a single-stranded overhang, to facilitate toehold-mediated strand displacement reactions [43]. Genelet circuits have the advantages of modularity and programmability for switch parameters, such as concentrations of switches and activators, which are analogues of weights and thresholds of neurons in artificial neural networks [38].

A bistable network is a dynamic system with two distinct stable equilibrium states [44], and it is often found in cellular networks requiring decision making processes such as cell cycle regulation, cellular differentiation [45], and apoptosis. The bistable network can be designed by genelets in two ways [39,42]: two switches can be connected in a mutually inhibiting configuration, or a single switch can be connected to activate its own transcription (Figure 3C). An oscillator circuit that produces periodic signals is another hallmark of basic circuit elements, and it is often found in cell signaling systems including genetic oscillation [46,47]. A synthetic oscillator was constructed using genelets with three different designs [48] (Figure 3D): a two-switch negative feedback oscillator that utilized activating and inhibiting connections (Design I); an amplified negative feedback oscillator that included an additional self-activating connection (Design II); and a three-switch ring oscillator with three inhibitory connections (Design III). The three designs shared the same basic architecture of overall negative feedback in the system. An amplified negative feedback oscillator (Design II) could potentially have four different phases, unlike a simpler oscillator (Design I), and the ring oscillator with an extra connection (Design III) featured a slower oscillation. The ability to construct different circuit motifs using genelets demonstrated the modularity and programmability of the system design. However, it remains a challenge to maintain circuit operation, such as oscillation, for an extended period of time in batch mode because of the exhaustion of nucleoside triphosphate (NTP) fuel, loss of enzyme activities, and build-up of incomplete RNA transcripts [48].

**Figure 4:**
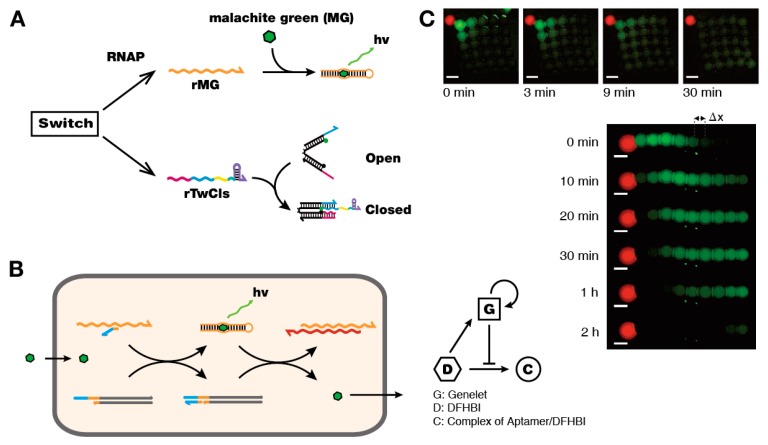
Extension of genelet circuits. (**A**) Driving downstream processes with genelet circuits. The output signal from genelet circuits can be functional RNA aptamers or can be used to drive DNA nanodevices such as DNA tweezers. (**B**) Signal propagation using encapsulated genelet circuits. Each droplet contains a genelet switch and aptamer-activator complex. Signal molecule (DFHBI) binds the aptamer and releases the DNA activator for the genelet switch. The activated genelet in turn produces kleptamer that binds the aptamer, releases DFHBI, and attenuates fluorescence output. (**C**) The experimental fluorescence images for one- and two-dimensional signal propagation. Reproduced with permission from [15,16].

In addition to providing basic motifs, these synthetic circuits could be coupled with downstream processes to dynamically control other molecular systems. A number of downstream processes, which can be considered as a downstream ‘load’, have been demonstrated including DNA-based nanomechanical devices (“DNA tweezers”) and functional RNA molecules (“aptamers”) [40] (Figure 4A). DNA tweezers have two rigid double-stranded “arms” connected by a single-stranded hinge, which can be opened and closed by nucleic acid inputs. Retroactivity of the load process degraded the upstream oscillator circuit, which was alleviated by introducing insulator circuits to prevent excessive consumption of core oscillator components and to amplify RNA signals.

Aptamers are nucleic acid molecules that fold into complex 3D shapes and bind to specific targets [49]. Functional RNA aptamers can be generated in vitro and tailored for a specific target, which are attractive features as downstream components to be controlled by genelet circuits. For instance, the transcription process can be monitored by using the aptamer against chromophore malachite green (MG) [40]. Sensing of specific molecules is enabled by designing the activator sequence of a genelet switch to bind to a specific aptamer, where the recognition of analyte by its aptamer releases the previously occupied DNA activator to activate the genelet. Using this approach, Dupin and Simmel demonstrated a genelet system to detect signal propagation in one and two dimensional array of compartments [50] (Figure 4B,C). The activity of enzymes can be controlled by using aptamers against T7 and SP6 RNAP, and an ssRNA/ssDNA with the complementary sequence of the aptamer, termed kleptamer, to provide yet another building block for synthetic biological circuitry with genelets [51]. These RNAP aptamers can also be used for logic circuits and transcriptional cascades [52].

Systems from natural processes and engineering disciplines provide further directions for developing genelet systems. Inspired by the architecture of electronic flip-flops, a genelet system design was proposed where the periods of a molecular clock were multiplied and divided [53]. Negative autoregulation provided model circuitry to produce outputs suitable for variable demands [54]. Adaptation in biological systems provided a framework to develop genelet circuits that detected fold-change of inputs [55]. Further, molecular titration utilized in natural and synthetic biological circuitry could be reiterated in genelet circuits with the support of mathematical modeling [56].

An analogous system, termed RTRACS (reverse transcription and transcription-based autonomous computing system), that relied on reverse transcriptase, DNA polymerase, RNAP, and RNase demonstrated modularity and programmability [57,58]. The modules of RTRACS received specific RNA input sequences and produced an RNA output through programmed computation. Experimental operation of an AND gate was demonstrated with RTRACS, and the prospect of more complex functionality such as oscillations was reported. The polymerase exonuclease nickase (PEN) toolbox bypassed the transcription step and relied exclusively on DNA and DNA-modifying enzymes to construct desired circuits [59,60]. Single-stranded DNA templates served as network architecture and short ssDNA species took the role of dynamic species that functioned as activators and inhibitors of templates. Despite its simplicity, the PEN toolbox successfully demonstrated bistability [60,61], oscillations [59,60,62], and pattern formations [63] through rational design approaches and easy monitoring [64]. An even more abstract approach is feasible with precisely programmed DNA sequences. Numerous studies demonstrated the power of DNA strand displacement circuits, including instructions, to create chemical reaction networks [65], logic circuits [66,67], neural networks [68,69], and oscillators [70] through toehold-mediated strand displacement [43]. These theoretical and experimental developments will enable future works to further enhance the programmability and complexity of synthetic in vitro circuits to control nucleic acid nanorobots for in vivo applications [71].

## 4. RNA Regulatory Circuits for Cell-Free Synthetic Biology

The programmable nature of RNA molecules that allows predictable design of structure and function provides a rationale to construct synthetic biological circuits with RNA toolkits. The most basic regulatory mechanism of RNA is to induce a trans interaction between the target mRNA and complementary RNA; RNAs that perform this function are called riboregulators [72]. Inspired by a plethora of natural examples of riboregulator-based gene expression control [73], synthetic biologists harnessed these design principles to create synthetic riboregulators in *E. coli* [74]. Following these seminal synthetic riboregulator systems, RNA-based synthetic biological circuits have emerged that are easily programmable with improved performance. In this section, we will discuss the recent progress in synthetic RNA regulators for cell-free diagnostic applications using toehold switch and small transcription activating RNA (STAR) as examples.

**Figure 5:**
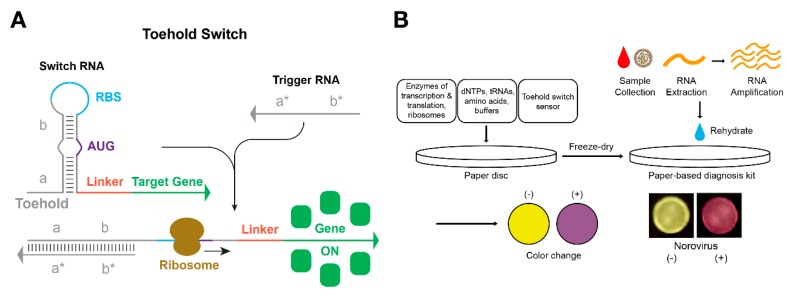
Toehold switch mechanism and application for paper-based diagnostics. (**A**) Mechanism of toehold switch. Linear-linear interaction between switch RNA and trigger RNA initiates from the toehold region. The resulting conformation of switch-trigger complex makes ribosome binding site (RBS) and start codon (AUG) available for ribosome access. (**B**) Freeze-dried paper-based diagnostic kit using toehold switch as a synthetic sensor. LacZ was used as a reporter gene so that the change of color could be checked by the naked eye.

A toehold switch is a de novo-designed regulatory RNA inspired by the mechanism of a conventional engineered riboregulator [75] (Figure 5A). In the switch RNA, the ribosome binding site (RBS) and the start codon are blocked by its own secondary structure. When the trigger RNA is introduced to initiate a toehold-mediated branch migration, a switch-trigger complex is formed in which the RBS and the start codon become available for the expression of the target gene. In *E. coli*, high-performance toehold switches showed dynamic ranges rivaling those of well-established protein regulators. This suggests toehold switches can provide a novel, high-performance platform for synthetic biological circuits. Moreover, the RBS and the start codon are not directly involved in base-pairing within the secondary structure design of switch RNA, which allows for the construction of a library of toehold switches without sequence constraints.

Capitalizing the functionality of toehold switches, Pardee et al. constructed a paper-based diagnostic platform using a toehold switch as a sensor [21] (Figure 5B). DNA that encoded the switch RNA and components for cell-free expression (enzymes, dNTPs, amino acids, etc.) were freeze-dried on paper discs, which could remain stable for storage at room temperature. Upon the addition of the trigger RNA specific to toehold switches, up to 350-fold induction of green fluorescent protein (GFP) was observed with the desired orthogonality. This laid the foundation for development of a paper-based diagnostic tool for Ebola virus by using toehold switches that specifically sensed part of nucleoprotein mRNA of Ebola as trigger RNAs [21]. β-galactosidase (LacZ) was used as a reporter gene to allow confirmation of results with the naked eye, and 24 toehold switches that targeted different sequences (Sudan strain 12 regions, Zaire strain 12 regions) were successfully tested. One notable feature was that the fold change of LacZ expression was dependent on the sequence of switch RNA, suggesting that the sequence design needed to be optimized for improved utility. In a follow-up study, Pardee et al. constructed a paper-based diagnostic tool for Zika viruses [35]. To improve sensitivity, a nucleic acid sequence-based amplification (NASBA) step was introduced to isothermally amplify the target region of Zika RNA. Their system responded normally to Zika virus but not to the closely related Dengue virus. In addition, they combined this system with a CRISPR/Cas9-based module to create a NASBA-CRISPR cleavage (NASBACC) system. This biosensor showed sophisticated diagnostic performance that could discriminate strains of Zika viruses (American or African) by utilizing the presence of the PAM sequence. In another recent work, Ma et al. demonstrated a paper-based cell-free diagnostic system that detected Norovirus with toehold switches [76] (Figure 5B). They introduced virus enrichment via synbody and α-complementation of LacZ enzyme to improve the sensitivity and speed of diagnosis. Takahashi et al. demonstrated a microbiota sensing system, rather than a single virus, with the same platform [77]. They designed switch RNAs based on the 16S rRNA sequence of each bacterial species, and functionality of the sensor was verified against 10 different bacterial strains. Moreover, they proposed the potential for paper-based diagnosis of more diverse target RNAs, including host biomarker mRNAs such as calprotectin, Interleukin 8, C-X-C motif chemokine ligand 5, oncostatin M, and specific pathogen toxin mRNA. These demonstrations provide evidence that toehold switches can be utilized as a generalizable platform for portable diagnostic systems in field testing diseases and other environmental samples.

To process an increasing complexity of inputs through synthetic biological circuitry, it is necessary to integrate a number of input signals in a seamless manner. To achieve this goal, the basic mechanism of the toehold switch has been expanded to incorporate multiple toehold switches in the same RNA transcript, which facilitates signal integration, termed a ‘ribocomputing’ strategy [78]. Green et al. implemented a complex logic system (combination of AND/OR/NOT) of 12 RNA inputs with five consecutive toehold switches colocalized in the same transcript. This 12-input logic circuit in *E. coli* provided evidence that a ribocomputing strategy could help in scaling up synthetic biological circuits in the future [79]. At the same time, novel RNA tools where translation is inactivated by trigger RNAs are also being explored. These include toehold repressor, three-way junction (3WJ) repressor, and looped antisense oligonucleotide, which enable a more complex and versatile logic with universal NAND and NOR gates [80,81].

**Figure 6:**
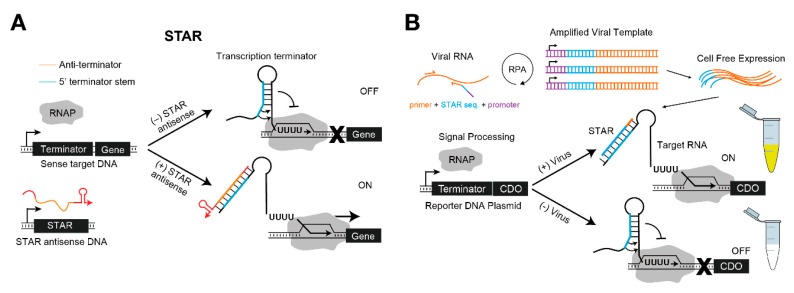
Small transcription activating RNA (STAR) system and application for detecting plant pathogens. (**A**) Mechanism of STAR. A transcription terminator consists of a stem-loop and a poly-U track, where the binding of STAR RNA breaks the step-loop structure such that transcription proceeds normally. (**B**) A platform to diagnose plant pathogens using STAR. Viral RNA in the sample can be amplified with the addition of a STAR sequence and a promoter through recombinase polymerase amplification (RPA). Then, the corresponding RNA transcribed through cell-free expression induces the expression of reporter gene (CDO, catechol 2,3-dioxygenase). RNAP: T7 RNA polymerase.

In a similar vein, Lucks et al. engineered the natural antisense RNA-mediated transcriptional attenuation mechanism of plasmid pT181, and they proposed an RNA toolkit that could turn off transcription [28]. Based on this, Takahashi and Lucks demonstrated that more diverse orthogonal transcriptional regulators could be designed by combining the module with natural antisense RNA regulators [82]. Building on these works, Chappell et al. devised a novel transcription regulatory system, termed the ‘small transcription activating RNA’ (STAR), which promoted transcription upon cognate trigger RNA binding [83] (Figure 6A). The natural mechanism was utilized in an opposite manner such that the complementary STAR RNA disrupted the transcription terminator structure of the target gene. In their first demonstration of the STAR system, the fold change ranged from 3- to 94-fold. In a subsequent work, they further optimized various domain lengths of STAR RNA through computational designs to create a STAR library with a broad fold activation range, from more than 400-fold to less than 10-fold, to allow for more sophisticated biological circuit designs [84].

A platform for plant pathogen detection was demonstrated using the STAR system [85] (Figure 6B). Verosloff et al. amplified viral DNA with the T7 RNAP promoter and an upstream STAR sequence by using recombinase polymerase amplification (RPA) with a primer that bound to a specific sequence of viral DNA. When viral RNA with the STAR sequence was transcribed by cell-free expression, the reporter RNA started normal transcription of catechol 2,3-dioxygenase (CDO) in the tube, which caused a colorimetric change in the sample observable with the naked eye. In particular, this system has the advantage that both the RPA and the cell-free expression steps are conducted isothermally using only body heat as a heat source.

A number of other works also demonstrated the utility of synthetic RNA regulatory parts for building synthetic biological circuits. Well-established anti-sense RNA (asRNA) could be utilized for translation regulation through binding at the 5′ untranslated region (UTR) and the start codon of the target mRNA [86]. Through analysis of the difference in repression of asRNA sequences, the repression was further enhanced by introducing the Hfq binding sequence into the asRNA [87]. Meanwhile, Rodrigo and Jaramillo developed a computational design tool, named ‘AutoBioCAD’, that allowed automatic RNA circuit design using secondary structure design and free energy analysis [88,89]. These RNA regulatory toolkits can contribute to the growing repertoire of cell-free synthetic biology applications including point-of-care devices for biomedical applications.

## 5. Encapsulation of in Vitro Circuits toward the Synthesis of Artificial Cells

In vitro synthetic biology has recently made progress towards realizing minimal cell systems. A key step toward this is the encapsulation of gene expression or TXTL systems in microscopic compartments. It has been demonstrated that both the kinetics and noise levels of chemical reactions are different in bulk than in cell-sized compartments or molecularly crowded solutions [90,91]. Thus, working with encapsulated synthetic biology components may improve our understanding of native cellular systems and how to emulate cell processes in synthetic systems [16]. Minimal cell systems can be designed to perform specified tasks autonomously, or they can network with native cells to increase sensing and actuation of biological systems. As encapsulation offers a barrier between critical components in synthetic circuits and surrounding environments, it is an essential step for designing effective minimal cell systems.

Both water-in-oil droplets and vesicles have been used to encapsulate gene expression systems in sizes relevant to cells (roughly 1–50 μm in diameter). Water-in-oil droplets have been demonstrated to be biocompatible, stable at high temperatures, and capable of withstanding deformation [92]. The oil medium surrounding the droplets greatly limits molecular exchange between individual droplets, effectively creating isolated, independent, cell-sized reaction chambers. Microfluidics can produce hundreds or thousands of uniformly sized droplets a minute, while shaken droplet protocols result in droplets of varying sizes.

Liposomes or vesicles more closely resemble native cells than water-in-oil droplets, but they are non-trivial to produce at cell sizes. Because the environment surrounding the vesicles is aqueous, exchange of biological molecules between the vesicles and the environment is possible for membrane-permeable molecules as well as membrane-impermeable molecules in the presence of surface pores or channels [93,94,95]. Emulsion transfer, thin-film hydration, and microfluidics have been shown to effectively encapsulate TXTL systems in vesicles [90,96,97,98,99,100]. Emulsion transfer and microfluidics allow for finer control of the vesicle size and contents than thin-film hydration techniques [100].

**Figure 7:**
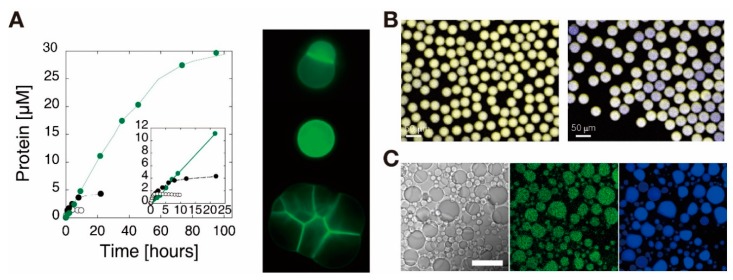
Synthetic gene circuits in cell-sized compartments. (**A**) (left) Expression of eGFP in bulk (open circles) and in a vesicle (closed dark circles), and expression of α-hemolysin-eGFP (closed green circles). (Inset) an expanded view of the first 20 h. (right) Fluorescence microscopy images of α-hemolysin-eGFP expressed in vesicles. Scale bar: 10 µm. Reproduced with permission from [16]. (**B**) Superimposed false-color images of cyan fluorescent protein (CFP) and yellow florescent protein (YFP) expressed in droplets without (left) or with (right) Ficoll. In the presence of Ficoll, the expression level is highly variable across the population of droplets. Reproduced with permission from [102]. (**C**) Microscopy images of mYPet expression inside an aqueous two-phase system (ATPS) water-in-oil droplets. The images of transmitted light, fluorescence microscopy of mYPet, and fluorescence microscopy of Alexa 647-labeled dextran are presented, from left to right. mYPet is preferentially expressed in the dextran phase rather than the polyethylene glycol (PEG) phase. Scale bar: 25 µm. Reproduced with permission from [103].

Early encapsulation of expression systems characterized the production of single reporter proteins in bulk and in vesicles. Noireaux and Libchaber reported that expression of GFP in both vesicles and bulk solution had similar durations and produced similar outputs [90] (Figure 7A). They showed that by expressing a GFP-labeled α-hemolysin pore, expression was increased by one order of magnitude. The toxin α-hemolysin acts as a pore in lipid bilayers with a molecular mass cutoff of 3 kDa, which allows nutrients from a surrounding feeding solution to enter the vesicle. Tan et al. demonstrated that vesicles protected an encapsulated GFP expression system from RNase A in the aqueous medium surrounding the droplet, which inhibited gene expression via degrading RNA [101].

Actualization of more complex gene expression systems, such as oscillators and cascading gene circuits, have subsequently been described in compartments [97,104]. In cascading reactions, products of an initial transcription system are necessary for further TXTL processes in the circuit. Garamella et al. encapsulated both five- and six-gene cascading circuits within vesicles [22]. They reported both an increase in the average expression across a population of 20–30 vesicles as well as an increase in the variability of expression for individual vesicles within the population for the six-gene circuit compared to the five-gene circuit. Adamala et al. reported that higher-order cascading circuits in vesicles produced similar amounts of protein to bulk reactions containing the same volume [105]. They observed smaller vesicles than the work by Garamella and colleagues, using mammalian HeLa cell extracts, and their circuits were triggered by diffusion of doxycycline through α-hemolysin pores in the membranes of the vesicles. The average expression for the population of vesicles containing a three-gene cascading circuit produced less fLuc than vesicles containing either one- or two-gene cascading circuits. The encapsulated three-gene circuit did, however, produce similar amounts of fLuc to a bulk solution with the same reaction volume, while the encapsulated one- and two-gene circuits produced less than the corresponding bulk reactions. Both reports noted that the high variability in expression for the higher-order circuits likely was due to nonuniform encapsulation of the individual components of the circuit throughout the population of vesicles. This phenomenon has been described in other works, and it even affects lower-order genetic circuits such as simple transcription of eGFP [106].

In addition to studying the effects of compartmentalization on TXTL systems, the influence of molecular crowding on encapsulated systems is relevant for both minimal cell design and understanding native cell processes. The crowded interiors of cells have been shown to influence intracellular reaction rates [107]. Hansen et al. investigated the relationship among stochasticity of expression within water-in-oil droplets, concentration of genes, and concentration of crowding molecules [102]. They reported that introduction of the crowding molecule Ficoll 70 resulted in microenvironments of cyan fluorescent protein (CFP) and yellow fluorescent protein (YFP) within the droplets for the duration of expression (Figure 7B). They concluded that the microenvironments formed because the rate of mRNA production was greater than the diffusion rate of the macromolecules involved in TXTL, as CFP and YFP diffused evenly through the droplets after the expression completed. They also noted that decreasing the available copies of gene within the expression system further increased the stochasticity of expression across the population of droplets. Tan et al. reported the effects of crowding molecules on minimal gene expression systems in vesicles, noting that crowding due to large dextran polymers increased expression of GFP in larger vesicles, while it had little effect on expression in smaller vesicles [101].

Molecular crowding not only affects reaction rates within cell-sized compartments but also leads to crowding-induced phase separation, which is another area of interest for synthetic biology efforts [108,109]. Liquid phase separation is a form of membrane-less partitioning, which occurs in native cell structures such as nucleoli and centrosomes, and is influenced by temperature, pH, concentration, and other factors [110]. Torre et al. showed confined expression of mYPet, a fluorescent protein, within a single phase of an aqueous two-phase system (ATPS) [103] (Figure 7C). The ATPS was a result of introducing both polyethylene glycol (PEG) and dextran to water-in-oil droplets, which separated into distinct PEG- and dextran-rich phases within the droplet. Torre et al. hypothesized that confinement of the gene expression to the dextran phases was a result of TXTL machinery partitioning the less hydrophobic dextran phase of the droplet. They reported no expression in encapsulated aqueous three-phase systems, suggesting this was due to splitting of the TXTL components between the dextran and Ficoll phases within the droplets.

**Figure 8:**
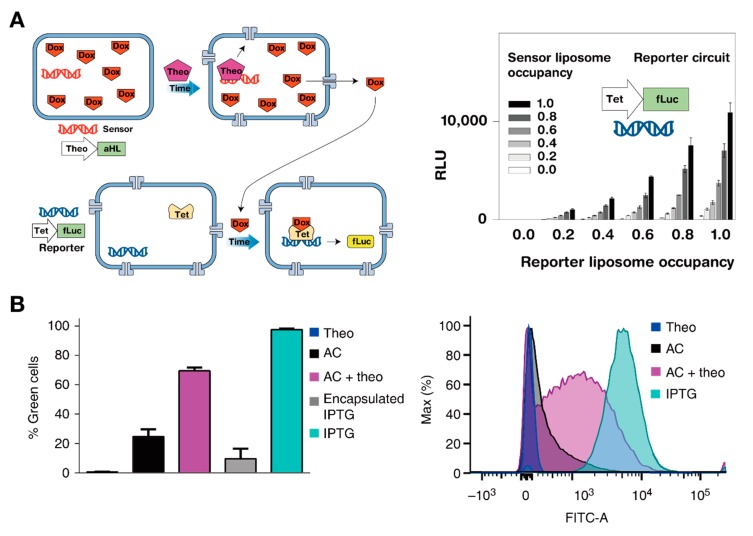
Interconnecting artificial and natural cells. (**A**) (left) Schematic design of synthetic sensor and reporter liposome pair, which contain bacterial and mammalian TXTL machinery, respectively. α-hemolysin (aHL) produced by theophylline treatment in the sensor liposome releases internal doxycycline to the environment, which in turn triggers expression of fLuc in the reporter liposome. (right) Expression of fLuc in different ratios of sensor and reporter liposomes. Occupancy refers to the ratio of droplets that contain TXTL machinery for both sensor and reporter droplets. Reproduced with permission from [105]. (**B**) (left) Flow cytometry data for *E. coli* containing a plasmid for GFP in the presence of the following components: theophylline (Theo), artificial cells (AC), artificial cells with theophylline (AC + Theo), isopropyl β-D-1-thiogalactopyranoside (IPTG) encapsulated in vesicles (Encapsulated IPTG), and IPTG in the bulk solution (IPTG). (right) Histogram of flow cytometry data shown in the left panel. Fluorescent signal is only increased in the presence of artificial cells and theophylline. Reproduced with permission from [115].

As the complexity of artificial cells increased, so too has the exploration of communication between networks made of artificial and native cells [50,111,112,113,114]. After showing that two distinct liposome-based minimal cells in a shared environment could respond to the same trigger without crosstalk, Adamala et al. demonstrated cascading networks of synthetic minimal cells as well as fusion-controlled TXTL systems [105] (Figure 8A). They realized a cascading expression system in which products from one vesicle that contained bacterial TX machinery triggered a response in a separate vesicle that contained mammalian TL machinery. Their work demonstrated that encapsulating TXTL systems provided modularity, allowing for interaction between otherwise incompatible components. Lentini et al. increased the sensing capacity of *E. coli* through networking with a synthetic translator cell [115] (Figure 8B). They induced expression of GFP within *E. coli* by creating an artificial cell that produced a chemical signal familiar to the bacteria, isopropyl β-D-1-thiogalactopyranoside (IPTG), in response to theophylline (Theo), which was otherwise undetectable to the native cell.

The exploration of artificial TXTL systems in cell-sized compartments is necessary for realizing artificial cells and fully understanding intracellular processes in confined and crowded environments. A number of complex gene expression circuits have been demonstrated in cell-sized compartments; however, the variety of components available for design—lipid bilayer vesicles vs water-in-oil droplets, bacterial vs mammalian cell-extracts, and so on—makes direct comparison between different studies difficult. The often encountered high variability in component concentrations during encapsulation processes may be alleviated through further exploration of encapsulation methods, fusion, and intercompartment exchange processes. Through compartmentalization, previously incompatible natural or synthetic systems in bulk solution can be interconnected as modular components, which paves the way for increasing the complexity of cell-free synthetic circuits and coordination with native cells.

## 6. Artificial DNA Structures and Systems for in Vitro Synthetic Biology

Because of its predictable self-assembly properties, DNA has been used to build versatile molecular machines and structures [116,117]. Complementary Watson–Crick base pairing between segments of synthetically designed DNA strands is utilized for the rational design of these static and dynamic devices, which can function autonomously by processing information obtained from its surroundings. DNA systems and circuits can present properties comparable to devices naturally found in the living cell, for example, it is possible to build DNA nanotubes with size and mechanical properties comparable to cytoskeletal filaments, DNA nanopores that dock on lipid bilayers with selective permeability, and transcriptional oscillators that could serve as clocks in synthetic cells [40,48,118,119]. DNA nanostructures could serve as physical components such as scaffolds, pores, and transport elements in artificial cells. Nucleic acid strand displacement reactions could be used to build sensors and signaling pathways [66,120,121,122]. Yet, these synthetic DNA systems may have difficulty in achieving desired structural integrity and functionality in the cellular environment since DNA nanostructures and networks have been typically characterized in buffer conditions very different from the complex environment of a cell. Therefore, it becomes a necessity to explore synthetic DNA systems in cell lysates, serum, and cell-free extracts to develop design rules for proper operation in complex cellular environments and to realize their full potential as programmable components for in vitro and in vivo synthetic biology.

**Figure 9:**
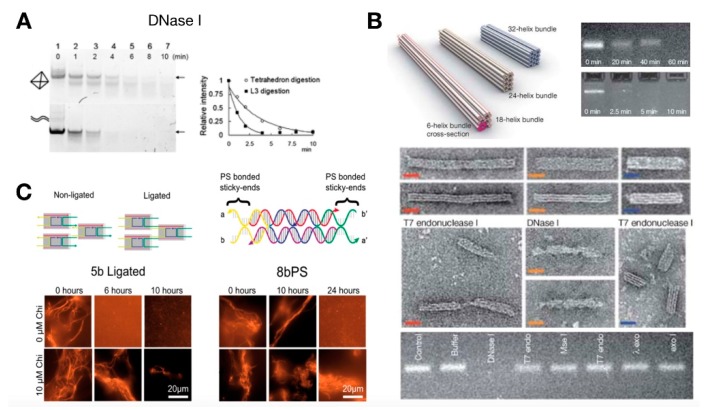
Characterization of DNA structures in cell-like media. (**A**) Denaturing polyacrylamide gel electrophoresis (PAGE) of tetrahedron and duplex DNAs with nonspecific degradation by DNase I. Digestion of the unligated tetrahedron is gradual and appears to generate a well-defined product, whereas digestion of linear DNA appears to be rapid and nonspecific. Reproduced with permission from [123]. (**B**) Stability of honeycomb-packed DNA nanostructure: 140 nm (18-helix bundle), 100 nm (24-helix bundle), and 70 nm (32-helix bundle), from left to right, were used for stability screening with TEM and agarose gel electrophoresis. Scale bar = 20 nm. Reproduced with permission from [124]. (**C**) Enhanced stability of DNA nanotubes with χ-site integration and chemical modifications in *E. coli* TXTL system. Fluorescence microscopy images of five-base DNA nanotubes with ligation of tile sticky ends and eight-base DNA nanotubes with phosphorothioate-bonded tile sticky ends incubated in TXTL with and without **χ**-site DNA present. Scale bar = 20 μm. Reproduced with permission from [126].

The presence of cytoplasmic enzymes can affect the structural stability of synthetic DNA systems. Kuem et al. measured the half-life of tetrahedral DNA nanostructures (TDNs) in the presence of DNase I and found that the stability of TDNs was more than twice that of double-stranded DNA [123] (Figure 9A). Castro et al. incubated DNA origami structures in the presence of different nucleases—DNase I, T7 endonuclease I, T7 exonuclease, *E. coli* exonuclease I, lambda exonuclease, and MseI restriction endonuclease [124]. Only DNase I and T7 endonuclease I were found to degrade the test origami structure, where the DNA origami structure could withstand complete degradation for 2 h in the presence of DNase I in contrast to the duplex plasmid DNA that disappeared within 5 min (Figure 9B). The interconnectivity and dense packing of the DNA nanostructures rendered some resistance to degradation by nucleases.

Another cell-like medium in which to characterize synthetic DNA systems can be cell lysates—the mixtures containing cellular components created by breaking down the membranes of cells. Mei et al. tested the stability of DNA origamis in cell lysate and reported that single- and double-stranded nucleic acids could not be recovered, whereas DNA origami could be recovered after up to 12 h [125]. However, the physiological relevance of this particular study was damped by the fact that cell lysate used sodium dodecyl sulfate (SDS) and deoxycholic acid (DCA), which suppressed many cellular enzymes. Therefore, a more physiological cell lysate preparation should be used for better assessing synthetic DNA systems in cell-like media.

A useful platform for rapid characterization of synthetic components is the *E. coli* cell-free TXTL system. TXTL reiterates the physiological conditions found in cells as well as harsh linear DNA degradation through the RecBCD complex. Klocke et al. tested the stability of tile-based DNA nanostructures in the TXTL system, demonstrating that the stability of structures increased significantly in the presence of χ-site double-stranded DNA, which was an inhibitor of the RecBCD complex [126] (Figure 9C). With the addition of 10 μM χ-sequences, tile-based nanotubes assembled from ligated DNA strands were stable in TXTL for more than 10 h. Further, phosphorothioation of the strands within nanotubes extended their viability in TXTL for more than 10 h without, and 24 h with, χ-sequences. However, chemically modified strands in DNA structures can introduce toxicity or trigger unwanted immune responses when introduced in cells [127]. Thus, chemical modifications should consider potential trade-offs of structural stability and cell toxicity.

**Figure 10:**
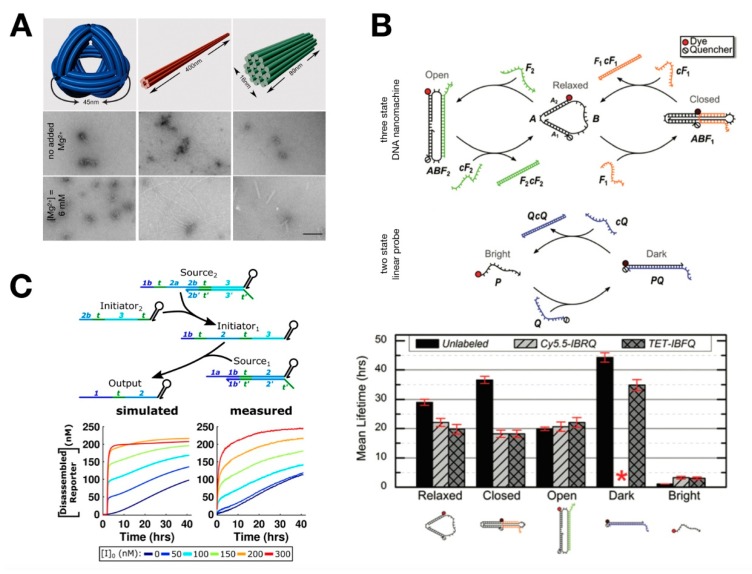
Characterization of DNA structures and circuits in serum. (**A**) 3D model of DNA nanooctahedron (DNO), six-helix bundle nanotube (NT), and 24-helix nanorod (NR) (top). TEM images of nanostructures incubated in unmodified (middle) or Mg^2+^-adjusted (bottom) medium. Structural integrity is maintained for all three designs with additional Mg ion. Scale bar = 100 nm. Reproduced with permission from [129]. (**B**) (top) Three-state DNA nanomachine transitions between relaxed, closed, and open states with the addition of fuel strands and their complements. The two-state linear probe transitions between bright and dark states upon hybridization of the dye-labeled probe strand, P, and the quencher-labeled strand, Q. (bottom) Mean lifetimes of the DNA nanomachines and linear probes show considerable differences in degradation rates. Reproduced with permission from [130]. (**C**) (top) Schematic of two-layer DNA cascade reaction. (bottom) Simulation results of a two-layer cascade with 5 bp toeholds using the fitted parameters and experimental measurements. Reproduced with permission from [133].

A number of studies were carried out to test the stability of DNA systems in serum and serum-supplemented media. The Sleiman group tested the stability of DNA assemblies in 10% fetal bovine serum (FBS) [128]. They reported that individual strands had a half-life of less than one hour, whereas the half-life of DNA structures in the shape of a triangular prism was closer to two hours. Hahn et al. tested the stability of DNA origamis in mammalian cell culture media supplemented with serum, and they indicated that DNA nanostructures were sensitive to depletion of Mg^2+^ in tissue culture medium [129] (Figure 10A). Interestingly, structural stability was significantly enhanced with the addition of actin, a protein that competitively binds to nucleases. No observable differences in cell growth, viability, or phenotype were present when actin was included in the medium.

The functionality of synthetic DNA circuitry is an important goal to achieve in the cellular environment. This spurred a number of studies on DNA circuitry in serum and serum-supplemented media. Goltry et al. investigated topological influences on the lifetimes of DNA devices using a three-state DNA tweezer nanomachine and a two-state linear probe in human serum and FBS [130] (Figure 10B). Degradation analysis revealed that the mean lifetimes of both systems in human serum were roughly six times longer than those in FBS. They reported that the device lifetimes varied greatly with topology (i.e., circular vs linear) and molecular conformation (i.e., shape of the structure), potentially providing a simple design rule to program structural stability or fragility. Graugnard et al. tested an autocatalytic strand-displacement network, reported by Zhang and colleagues [131], in human serum and mouse serum [132]. With the addition of SDS to halt nuclease activity, the synthetic network was functional in serum with both DNA and RNA catalysts. Fern and Schulman investigated strategies to enable strand-displacement circuits to operate in 10% FBS [133] (Figure 10C). By inhibiting nuclease activity using actin protein, and by modifying DNA complexes with hairpin extensions on the 3′ ends of DNA strands, the half-life of DNA strands increased by 10-fold. Through these modifications, a multilayer cascade circuit was demonstrated that released a desired output strand with controlled kinetics with the aid of computational modeling.

Taken together, densely packed and interconnected DNA nanostructures, such as DNA origami, are consistently more stable than structurally simple nucleic acid architectures in cell-like environments. Nucleases can be a primary cause for structural instability of synthetic DNA systems; however, other processes also need to be taken into consideration. For instance, nonspecific transcription by RNAP can produce transcripts that, in turn, can interact with DNA nanostructures, leading to disassembly via a toehold-mediated branch migration [134]. Thus, more systematic research is warranted to develop strategies that shield DNA systems from unintended crosstalk with biological components and that maintain integrity of devices within the cellular context. Use of actin or χ-sites as molecular decoys, structural modifications to increase interconnectivity, chemical modifications, and hairpin extensions are some of the strategies explored towards achieving better functionality of synthetic DNA systems. The improved design rules for DNA nanomachines and circuits may support the translation of devices operational in cell-free settings to the cellular environment.

## 7. Mathematical Modeling Supports the Development and Analysis of in Vitro Systems

Mathematical modeling has contributed to the success of synthetic biology since its inception [1,2]. Models are helpful to support the design of synthetic systems and to explain quantitatively observed phenomena, which may be otherwise difficult to understand, especially when they include feedback loops. Validated models are also useful to make predictions and guide experiments, making it possible to save time and costly reagents. Ordinary differential equations (ODEs) are one of the simplest approaches to build mathematical models to describe kinetic systems. ODE models are particularly well-suited to capture systems operating at high copy numbers, so they are an excellent choice for in vitro synthetic biology. Because in an in vitro setting it is usually possible to collect a large amount of kinetic data in which experimental conditions are varied systematically, ODEs can be easily fitted to the data and yield solid estimates of various parameters that govern the kinetics.

**Figure 11:**
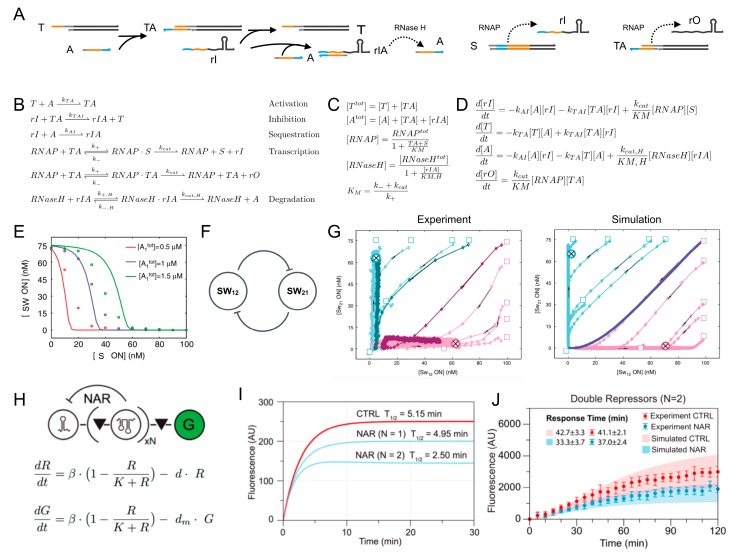
Ordinary differential equation (ODE) models for design and characterization of in vitro synthetic systems. (**A**) Schematic of an inhibited transcriptional switch. (**B**) List of reactions describing a transcriptional switch that is inhibited by RNA transcribed by a source template S. (**C**) Mass conservation and Michaelis–Menten expressions allow a simplification of the ODEs. (**D**) ODEs describing the inhibited transcriptional switch. (**E**) Example data showing the input–output steady state curve mapping the concentration of inhibitor source to the output switch concentration, overlapped with simulated steady state data (solid lines). Reproduced with permission from [39]. (**F**) A bistable switch can be constructed by interconnecting two inhibitor switches. (**G**) Experimental data (left) compared to simulated trajectories (right) of the bistable switch. Reproduced with permission from [39]. (**H**) Example transcription regulator used to build a gene performing NAR. G indicates GFP and its qualitative ODE model. (**I**) The qualitative ODE model suggests that the NAR circuit operates better when using at least two transcription repressors in tandem. (**J**) Simulations of detailed mechanistic models reproduce experimental data well. Adapted with permission from [32].

Many in vitro synthetic systems have been quantitatively modeled using ODEs that can be built systematically starting from a list of relevant chemical reactions. Transcriptional networks, for example, include synthetic genes (genelets), two enzymes, and mRNA species to create regulatory interconnections between genelets in a rational manner [39,48]. To formulate an ODE model that captures the kinetics of an inhibited genelet, the species to be considered are the template *T* (active and inactive), its DNA activator *A*, the RNA inhibitor *rI*, and RNAP and RNase H that control RNA production and degradation. RNA inhibitor is produced by a “source” template (S), whose concentration is constant. The active template *TA* produces an RNA output rO. The template and its activator are referred to as a “switch” (SW). The complete set of reactions associated with this system is shown in Figure 11A. Using the law of mass action, it is possible to write ODEs that describe the reaction kinetics. For example, the free template concentration *T* is converted to active template *TA* by binding to the activator *A* at rate constant *k_TA_*; in turn, the active template *TA* is converted back to the free template *T* when it interacts with the inhibitor *rI*, which displaces the activator at rate *k_TAI_*. As a consequence, we can immediately write the kinetics of the free template as:

(1) $$\frac{dT}{dt}=-k_{TA}\left[T\right]\left[A\right]+k_{TAI}\left[TA\right]\left[rI\right].$$

The ODEs for all other species can be derived with the same procedure. Because the total template concentration remains constant, then $\left[TA\right]=\left[T^{tot}\right]-\left[T\right]$, which means the model does not require a specific ODE for the kinetics of *TA*. For enzyme kinetics, it is possible to use the well-known Michaelis–Menten quasi-steady state approximation so that the available concentrations of RNAP and RNase H can be expressed with an analytical, static formula as a function of their substrate (Figure 11C). The complete ODE model is pictured in Figure 11D. This model can be fitted to kinetic data, and it reproduces the steady state input–output map of the inhibitable switch (the input is the concentration of source S, the output is the fraction of active switch) (Figure 11E).

More complex transcriptional networks can be modeled with the same approach by modularly composing the models of individual switches. For instance, an ODE model of a bistable switch (Figure 11F) could be immediately built by interconnecting the models of two inhibitable switches whose RNA outputs mutually inhibited transcription [39]. The models were augmented by taking into account undesired or putative reactions, such as transcription from inactive template T, and captured very well the kinetic experiments, as shown in Figure 11G. Similarly, Kim and Winfree developed [48] ODE models for different versions of a transcriptional oscillator, in which side reactions played a very important role. For example, the ability of the model to reproduce the oscillator kinetics (in particular the damping rate) was significantly improved by including incomplete degradation products that accumulated during the oscillator reaction and their potential interactions with activation and inhibition of the genelets. ODE models built using the law of mass action can be used to model genelets, molecular machines, and other molecular processes, and they can be used to computationally test the influence of new, modified, or unknown components on the system [40,135]. To summarize, mechanistic ODE models are successful at recapitulating the dynamic behaviors of in vitro synthetic systems, and they can be easily expanded to include additional species or reactions. Yet, these models can become very large, even in systems with few desired interactions, and obtaining physically meaningful fitted estimates for the model parameters requires the inclusion of tight bounds.

A phenomenological approach to building ODE models is advantageous in building models with few variables and parameters, which helps in obtaining more intuitive results on the behavior of the system under consideration. Rather than being built from a list of chemical reactions, phenomenological models rely on qualitative relationships between species. For example, the steady state behavior of the inhibitable genelet shown in Figure 11E could be modeled using a single ODE in which the source template could cause a decrease of active switch via a Hill-type function. Beyond transcriptional circuits, phenomenological models have been used for many synthetic systems built in vitro, including RNA regulator-based circuits [32,136,137]. Figure 11H shows the qualitative model for a transcription regulator that achieves negative autoregulation (NAR) [32]; the species in the equations are the concentrations of RNA (R) and GFP (G), whose productions decrease as the concentration of RNA (R) increases (self-inhibition). Using parameters from the literature, this simple model was used to compare the efficiency of one versus two tandem repressors, and it yielded the trajectories in Figure 11I that qualitatively agreed with the experimental data in Figure 11J. Although a detailed mechanistic model was required to quantitatively reproduce data (Figure 11J), the simple model provided useful insights on the system kinetics.

Limited modeling efforts have been dedicated to compartmentalized cell-free circuits, largely because this research is still in its early stages. Encapsulation can introduce noise and stochastic phenomena even when operating with few components at high concentration. The operation of a transcriptional oscillator, for example, was significantly affected by partitioning noise when encapsulated in water-in-oil droplets [104]; a model combining ODEs and stochastic partitioning of components (following a Poisson process) was able to recapitulate the variability in the circuit dynamics. Stochastic simulations could improve our understanding of noise observed in recent works aimed at encapsulation in high-order synthetic circuits [105,111,138].

## 8. Concluding Remarks

As synthetic biological systems have become larger and more complex, deciphering the intricate interaction of synthetic systems and biological entities becomes a challenging task. Cell-free synthetic biological approaches, with the aid of rapid progress in its scope, and toolkits may provide the right platform for rapid design–build–test cycles. New technological breakthroughs for synthetic biology, such as CRISPR-Cas systems, can also be elucidated in this simplified TXTL test bed [23]. The ease with which to program nucleic acids has dramatically accelerated the structural and functional complexity of nucleic acid-based molecular devices. These new developments encompass simplified synthetic model dynamical systems and nucleic acid nanostructures, as well as synthetic RNA regulatory components, which form the core of practical tools for biomedical applications. Compartmentalization for synthetic cells opens up ways for scientific inquiry and enhanced functionality through networks of synthetic and natural systems. Data-driven model building needs to guide the research and development towards complex synthetic systems with prescribed dynamics in the future. In the coming years, we anticipate that the utility of cell-free synthetic biology will rapidly expand the scope of biotechnology and synthetic biology, and it will provide innovative solutions in biomanufacturing therapeutics for biomedical applications and biologic products for industrial applications.
